# Supplementary material for: Multi‐dataset identification of innovative feature genes and molecular mechanisms in keratoconus
Source: J Cell Mol Med. 2024 Sep 19;28(18):e70079. doi: 10.1111/jcmm.70079 (PMC11412914; doi:10.1111/jcmm.70079)
Supplement: Supplementary file 2 — Material S2. [file JCMM-28-e70079-s002.zip › 7.GSEA analysis/5.RNF217-AS1/KEGG_PANTOTHENATE_AND_COA_BIOSYNTHESIS.html]

Details for gene set KEGG\_PANTOTHENATE\_AND\_COA\_BIOSYNTHESIS[GSEA]

|  || Dataset | RNF217-AS1.group.cls#h\_versus\_l |
| Phenotype | group.cls#h\_versus\_l |
| Upregulated in class | l |
| GeneSet | KEGG\_PANTOTHENATE\_AND\_COA\_BIOSYNTHESIS |
| Enrichment Score (ES) | -0.52992463 |
| Normalized Enrichment Score (NES) | -1.3471427 |
| Nominal p-value | 0.13530655 |
| FDR q-value | 0.38329437 |
| FWER p-Value | 0.917 |
Table: GSEA Results Summary

  

Fig 1: Enrichment plot: KEGG\_PANTOTHENATE\_AND\_COA\_BIOSYNTHESIS      
 Profile of the Running ES Score & Positions of GeneSet Members on the Rank Ordered List

  

| SYMBOL | TITLE | RANK IN GENE LIST | RANK METRIC SCORE | RUNNING ES | CORE ENRICHMENT || 1 | UPB1 | na | 4405 | 0.237 | -0.0377 | No |
| 2 | DPYS | na | 9307 | 0.099 | -0.1417 | No |
| 3 | VNN1 | na | 9613 | 0.093 | -0.1156 | No |
| 4 | ENPP3 | na | 10455 | 0.079 | -0.1102 | No |
| 5 | PPCS | na | 11663 | 0.060 | -0.1224 | No |
| 6 | PANK2 | na | 14129 | 0.029 | -0.1825 | No |
| 7 | PANK3 | na | 16878 | -0.000 | -0.2616 | No |
| 8 | COASY | na | 20863 | -0.038 | -0.3619 | No |
| 9 | BCAT2 | na | 25174 | -0.093 | -0.4511 | Yes |
| 10 | VNN2 | na | 25500 | -0.098 | -0.4235 | Yes |
| 11 | PANK1 | na | 26510 | -0.117 | -0.4088 | Yes |
| 12 | PANK4 | na | 27073 | -0.127 | -0.3771 | Yes |
| 13 | PPCDC | na | 32378 | -0.316 | -0.4114 | Yes |
| 14 | BCAT1 | na | 32474 | -0.323 | -0.2930 | Yes |
| 15 | DPYD | na | 33263 | -0.381 | -0.1726 | Yes |
| 16 | ENPP1 | na | 34337 | -0.572 | 0.0113 | Yes |
Table: GSEA details [plain text format]

  

Fig 2: KEGG\_PANTOTHENATE\_AND\_COA\_BIOSYNTHESIS      
 Blue-Pink O' Gram in the Space of the Analyzed GeneSet

  

Fig 3: KEGG\_PANTOTHENATE\_AND\_COA\_BIOSYNTHESIS: Random ES distribution      
 Gene set null distribution of ES for **KEGG\_PANTOTHENATE\_AND\_COA\_BIOSYNTHESIS**
